# Global burden trends and future predictions of ischemic heart disease attributable to air pollution in people aged 60 years and older, 1990–2021

**DOI:** 10.3389/fpubh.2025.1598092

**Published:** 2025-07-04

**Authors:** Yuanqin Zhao, Lihui Liu, Wei Fan, Man Qi, Bin Liao

**Affiliations:** ^1^Department of Cardiovascular Surgery, The Affiliated Hospital, Southwest Medical University, Metabolic Vascular Diseases Key Laboratory of Sichuan Province, Key Laboratory of Cardiovascular Remodeling and Dysfunction, Luzhou, China; ^2^Key Laboratory of Medical Electrophysiology, Ministry of Education and Medical Electrophysiological Key Laboratory of Sichuan Province, (Collaborative Innovation Center for Prevention of Cardiovascular Diseases), Institute of Cardiovascular Research, Southwest Medical University, Luzhou, China; ^3^Chinese PLA General Hospital, College of Pulmonary and Critical Care Medicine, Beijing, China; ^4^State Key Laboratory of Cardiovascular Disease, National Center for Cardiovascular Diseases, Chinese Academy of Medical Sciences and Peking Union Medical College, Fuwai Hospital, Beijing, China

**Keywords:** older adult population, global burden of disease, ischemic heart disease, air pollution, risk factor

## Abstract

**Background:**

Ischemic heart disease (IHD) is one of the leading causes of mortality and disability among the older adults. Studies have shown that air pollution (AP) exacerbates the risk of cardiovascular diseases, particularly IHD, posing significant health threats to older adults and increasing disease burden. Recently, with the rapid advancement of industrial technology, environmental pollution has become increasingly prominent. Therefore, it is of utmost significance to investigate the impact of AP on IHD burden, especially on vulnerable populations such as older individuals.

**Methodology:**

Global Burden of Disease (GBD) Study 2021 data was used to analyze and quantify contemporary global burden of IHD in individuals aged 60 and above due to AP and for trends for the time period between 1990 and 2021 using disease burden indicators such as deaths, DALYs, YLDs, YLLs and corresponding age-standardized rates (ASRs). Specifically, we are in patterns of disease burden of IHD in various subgroups stratified by age, sex, Sociodemographic Index (SDI), and GBD regions and countries. SDI-based analyses were conducted to explore the association between socioeconomic development and IHD burden attributable to AP. In addition, we employed the Bayesian Age-Period-Cohort (BAPC) model to provide future estimates of IHD burden attributable to AP for persons aged ≥60. This study provides a comprehensive overview of disease burden patterns of AP-related IHD from multiple perspectives.

**Results:**

Between 1990 and 2021, global IHD burden attributable to AP in individuals aged 60 and above rose in terms of deaths, DALYs, YLDs and YLL, while there was a decline in ASRs. Overall, the disease burden in the older adult population remains high, with older age groups experiencing the greatest burden and the most significant decrease in ASRs. Men consistently faced a higher burden than women. Except for high-SDI regions, the burden increased across all other SDI regions, while ASRs declined across all SDI categories. Regions such as East Asia and South Asia significantly contributed to the global burden, with marked regional differences in ASRs. High-income regions saw a more pronounced decrease in ASRs, whereas low-SDI regions, such as East Asia and South Asia, exhibited slower reductions. China and India together account for over half of the global burden. ASR of IHD attributable to AP generally decreased as SDI increased. Projections for 2036 suggest that the disease burden will continue to rise, while ASRs will gradually decline, with men continuing to bear a higher burden than women.

**Conclusion:**

Although the ASRs of IHD due to AP in individuals aged 60 and older have generally decreased, the absolute number of cases continues to rise. The burden of IHD varies significantly across different genders, age groups, GBD regions, SDI regions, and countries, with older age groups and males exhibiting higher ASRs. In GBD regions such as East Asia and South Asia, as well as in low-SDI regions and developing countries, inadequate healthcare infrastructure and limited AP control exacerbate the impact of AP, resulting in a disproportionately heavy burden. As the global older adult population continues to grow, the health risks associated with AP-induced IHD are expected to worsen, posing an increasing public health challenge. Public health policies should reduce exposure to AP in men and address lifestyle-related factors in this group. In disease-burdened areas, health care systems need to be improved, air quality control policies need to be strengthened, and more efficient health management practices for older individuals need to be adopted. Public health awareness and informing individuals about risks of AP are also necessary for avoiding future disease burdens. Projections for 2036 are for a significant increase in IHD cases and global public health policies should be directed towards reducing AP and developing efficient health care infrastructure to address future challenges.

## 1 Introduction

Ischaemic heart disease (IHD), or coronary artery disease, is a condition resulting from a reduction in blood flow to the myocardium as a direct consequence of compromised coronary circulation. It is predominantly characterized by myocardial ischaemia and consequential myocardial injury (1). The coronary arteries are responsible for delivering the myocardium with a supply of oxygen and nutrients. Narrowing or occlusion of these coronary arteries is usually responsible for IHD development. Atherosclerosis, thrombosis, and spasm of coronary arteries are common aetiological causes (2). Incidence and mortality rates for IHD increase steeply with age and turn older individuals into a high-risk population for ischemic heart disease (3). Global population aging has become a central demographic characteristic of the 21st century and is a hallmark of developed and some developing nations with a rapidly expanding proportion of older individuals aged 60 and above (4). The World Health Organization has estimated that there will be 1 billion people aged 60 and older in 2020, which is projected to rise to 1.4 billion in 2030 and to 2.1 billion in 2050 (5). With growing population aging, IHD has become a leading cause of death and disease burden across the world and is imposing an unprecedented burden on public health systems and adding disease burden to individuals and societies (6).

But with industrialisation and social modernisation, air pollution (AP) has emerged as a focal point for global public health. AP has become the fourth cause of death and disability-adjusted life years (DALYs) worldwide, according to the 2019 Global Burden of Disease study (7), AP causes about 7 million deaths each year and is responsible for 12% of all deaths worldwide and contributes to global cardiovascular disease burden overall (8). Current epidemiological studies reveal that exposure to air pollutants such as fine particulate matter (PM2.5) and other air pollutants has been found to induce chronic inflammation and endothelial dysfunction and enhance cardiovascular disease mortality by a significant margin by means of coronary atherosclerosis and ensuing ischemic heart disease (9–11). On a whole, AP-related factors are of particular significance for older individuals.

The aim of this study is to investigate the disease burden of IHD attributed to AP with a focus on individuals aged 60 and above. From recent estimates of the 2021 Global Burden of Disease (GBD), we will evaluate and compare the worldwide disease burden of IHD attributed to AP and trends for the period between 1990 and 2021. The analysis will make use of indicators such as deaths, DALYs, years lived with disability (YLDs), years of life lost due to premature death (YLLs) and corresponding age-standardized rates (ASRs). The study will also identify disease burden by different subgroups with respect to gender, age, Socio-Demographic Index (SDI), and GBD regions and countries. We will also apply the Bayesian Age-Period-Cohort (BAPC) model to make future projections of disease burden of IHD caused by AP for individuals aged 60 and above. This detailed breakdown will uncover insights regarding the nature of disease burden caused by AP.

## 2 Methods

### 2.1 Data acquisition

GBD 2021 data are employed in this study to approximate the worldwide burden of ischemic heart disease due to AP risk factor in people aged 60 and older. The GBD 2021 estimates are publicly available through the Global Health Data Exchange (GHDx) platform,^1^ providing open access to detailed global and regional health indicators. GBD 2021 provides estimates for 371 diseases and injuries, covering incidence, prevalence, deaths, YLDs, YLLs and DALYs. Additionally, it includes 88 risk factors related to behavioral, environmental, occupational, and metabolic domains. The calculation methods of YLLs, YLDs, and DALYs have been described in other literature (12), but in short, YLLs were calculated by multiplying the number of deaths at each age by the standard life expectancy at that age, based on the reference life table used in GBD 2021 (13), and YLDs were estimated by multiplying the prevalence of each sequela by its corresponding disability weight, reflecting the severity of health loss associated with that condition (14). DALYs, a composite metric of disease burden, were calculated as the sum of YLLs and YLDs, capturing both premature mortality and non-fatal health outcomes (15). The Social Development Index (SDI) is a composite measure of socioeconomic development, incorporating total fertility rates, average years of schooling, and lagged per capita income for populations aged 15 and older. Countries and regions are classified into five SDI groups—high, middle-high, middle, low-middle, and low—based on SDI scores ranging from 1 to 0 (16). GBD 2021 classifies 204 countries and territories into seven super-regions, which are further divided into 21 regions based on geographic proximity and epidemiological characteristics (17). This study analyzes data from 204 countries and regions between 1990 and 2021. The report provides a comprehensive trend analysis and assessment of the global burden of IHD attributable to AP in people aged 60 and older.

### 2.2 Data filtering

The dataset was filtered to include only indicators relevant to the impact of AP on ischemic heart disease in individuals aged 60 years and older. Primary variable indicator included: measure (deaths, YLDs, YLLs and DALYs), sex subgroups (both sexes, females and males), age subgroups (60–64, 65–69, 70–74, 75–79, 80–84, 85–89, 90–94, and 95 + years), location subgroups 1 (five SDI groups), location subgroups 2 (seven super-regions, further divided into 21 regions), location subgroups 3 (204 countries) metric (number, rate and percent), risk factor (air pollution), and time period (1990–2021).

To evaluate temporal trends, the estimated annual percentage change (EAPC) was calculated using a log-linear regression model. A new categorical variable, direction, was introduced to represent whether the measure (deaths, YLDs, YLLs and DALYs) trend was increasing or decreasing over time based on the EAPC value.

### 2.3 EAPC

Temporal trends in deaths, DALYs, YLDs, and YLLs attributable to air pollution were assessed for different geographic units using the EAPC. The EAPC represents the average annual percentage change in age-standardized rates (ASRs), quantifying the temporal trend over the study period (18–20).

A linear regression model was applied to estimate EAPC, with ASR as the dependent variable and calendar year (*t*) as the independent variable:


ln(ASR)=α+βt+ε

where *α* is the intercept, *β* represents the annual rate of change in ASRs, and *ε* is the error term (19, 21, 22).

The EAPC was then calculated as follows:


$$\mathrm{EAP}C_{\text{with}\;95\%\mathrm{CI}}=100\times (e^{\beta }-1)$$

where 𝛽 is the slope obtained from the linear regression of the natural logarithm of ASR. with 95% confidence intervals (CIs) used to determine the statistical significance of the trends.

### 2.4 SDI analysis

SDI categories were used to compare the disease burden across different levels of socioeconomic development. The relationship between SDI and the burden of IHD attributable to AP was examined by calculating SDI-specific disease death rates (23). The `ggplot2` and `ggrepel` packages in R were employed for data manipulation and visualization.

### 2.5 Future projections (2022 to 2036)

For better public health policy-making and medical resource distribution, we stratified the population by gender subgroups (both sexes, females and males) and used the Bayesian-Aperiodic-People-Cohort (BAPC) model to predict future trends in deaths, YLLs, YLDs, and DALYs for IHD for the next 15 years. By including age-specific trends and time-related changes, these models provide a credible and detailed projection of future IHD burden (24–26). Studies have found that it is possible to make effective approximations of marginal posterior distributions by combining the Integrated Nested Laplace Approximation (INLA) with the BAPC model and avoiding mixing and convergence issues that are typical of the Markov Chain Monte Carlo (MCMC) sampling method used by traditional methods. This model provides flexible, accurate, and computationally efficient projections, making it well-suited for analyzing long-term trends in chronic diseases and environmental health risks (27). BAPC model is appropriate for studying environmental risk factor (such as AP), chronic diseases (such as IHD) and health trends in specific populations (such as people aged 60 years and older).

### 2.6 Statistical analysis

All statistical analyses and data visualizations were performed using R (version 4.3.2). Descriptive statistics were generated for all key variables, and results were expressed as means with 95% CIs. For trend analyses, *p*-values < 0.05 were considered statistically significant. We generated data visualizations, including bi-lateral and two-axis plots, using the ‘ggplot2’ and ‘Benchmarking’ packages in R.

## 3 Results

### 3.1 Analysis of the global burden of disease for IHD attributed to AP in individuals aged over 60 and the trend of change from 1990 to 2021

**Figure 1:**
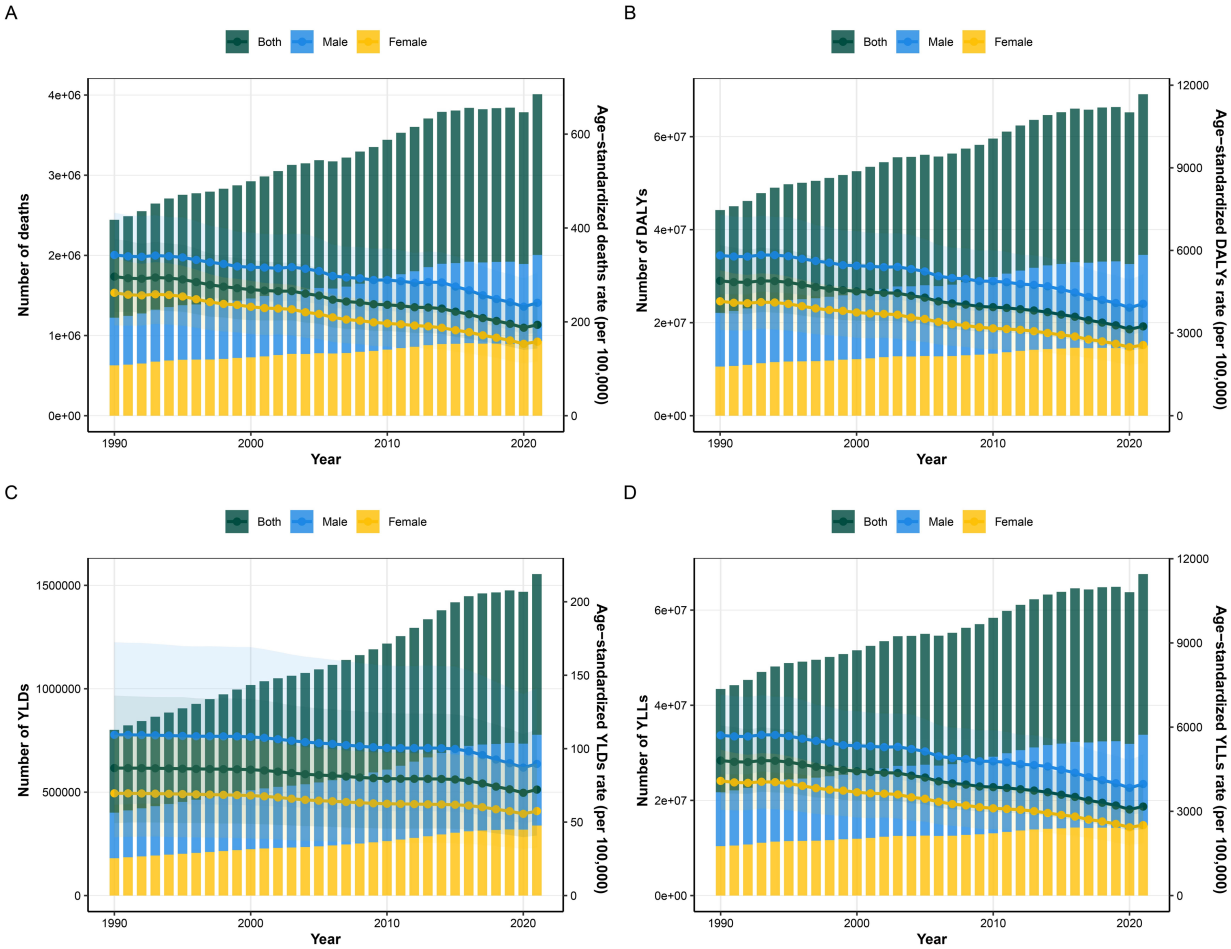
Temporal trends of various indicators over different years, reflecting the impact of air pollution on ischemic heart disease in people aged 60 years and older by sex subgroups from 1990 to 2021. The left Y-axis represents the number of the total disease burden attributable to each indicator, The right Y-axis represents the age-standardized rate of the total disease burden attributable to each indicator, whereas the X-axis represents the years. DALYs-disability-adjusted life years, YLDs-years lived with disability, YLLs-years years of life lost. The shaded area represents the 95% confidence interval (CI) of the age-standardized rate.

Between 1990 and 2021, IHD burden attributable to AP in individuals aged above 60 years presented a rising trend in Disability-Adjusted Life Years (DALYs), deaths, Years Lived with Disability (YLDs), and Years of Life Lost (YLLs). Conversely, the corresponding Age-Standardized Rates (ASRs) demonstrated a downward trend (Figure 1). By 2021, the global figures for DALYs, deaths, YLDs, and YLLs due to AP-related IHD were 34,564,173 (95% Uncertainty Interval [UI]: 25,887,530 to 43,130,304), 2,005,125 (95% UI: 1,484,963 to 2,514,494), 777,285 (95% UI: 440,728 to 1,219,129), and 33,786,889 (95% UI: 25,257,005 to 42,183,966), respectively. The corresponding ASRs were 3,242.22 (95% UI: 2,424.71 to 4,048.51), 193.65 (95% UI: 143.12 to 243.06), 72.13 (95% UI: 40.91 to 113.06), and 3,170.09 (95% UI: 2,366.06 to 3,960.79). These figures represent an increase of 56.3, 64.1, 94, and 55.6%, respectively, compared to 1990. The estimated annual percentage changes (EAPC) were −1.41 (95% CI: −1.50 to −1.31), −1.41 (95% CI: −1.51 to −1.31), −0.6 (95% CI: −0.68 to −0.53), and −1.42 (95% CI: −1.52 to −1.32), respectively (Tables 1–4).

**Table 1:** The DALYs cases and age-standardized DALYs rate of IHD attributed to air pollution in 1990 and 2021, along with their temporal trend.

Region	Rate per 100 000(95%UI)				
	1990		2021		1990–2021
	DALYs cases	The age-standardized DALYs rate	DALYs cases	The age-standardized DALYs rate	EAPC
Global	22107988 (16903345 to 27803896)	4895.95 (3716.01 to 6187.07)	34564173 (25887530 to 43130304)	3242.22 (2424.71 to 4048.51)	−1.41 (−1.5 to −1.31)
SDI region					
High SDI	4390759 (2705124 to 6182362)	3058.69 (1879.87 to 4313.35)	2040754 (1341896 to 2770967)	689.95 (456.59 to 934.87)	−5.12 (−5.23 to −5.01)
High-middle SDI	6530351 (4660856 to 8614951)	5820.33 (4129.53 to 7695.42)	7405535 (5275594 to 9674445)	2972.71 (2116.56 to 3882.58)	−2.45 (−2.73 to −2.17)
Middle SDI	5322710 (4168043 to 6534869)	5062.06 (3947.46 to 6216.48)	12231343 (9080868 to 15415001)	3988.26 (2956.56 to 5025.44)	−0.67 (−0.84 to −0.5)
Low-middle SDI	4322387 (3405288 to 5297568)	6610.63 (5198.62 to 8108.88)	9761841 (7525052 to 11964842)	6016.45 (4629.56 to 7379.96)	−0.22 (−0.3 to −0.14)
Low SDI	1504487 (1187060 to 1871229)	6303.99 (4962.2 to 7833.27)	3095481 (2417851 to 3791725)	5949.3 (4635.25 to 7291.38)	−0.11 (−0.2 to −0.02)
GBD region					
Andean Latin America	98550 (73960 to 125317)	4383.15 (3290.59 to 5567.1)	113278 (74222 to 160913)	1605.83 (1052.13 to 2280.29)	−3.71 (−3.98 to −3.44)
Australasia	32975 (1134 to 89348)	1093.85 (37.68 to 2966.78)	26117 (14012 to 39878)	340.74 (182.83 to 520.27)	−4.52 (−5.06 to −3.97)
Caribbean	157115 (100638 to 233977)	5091.57 (3241.46 to 7599.8)	207951 (130513 to 299026)	3080.23 (1935.25 to 4425.53)	−1.6 (−1.83 to −1.38)
Central Asia	521604 (327117 to 750823)	9991.17 (6266.67 to 14378.09)	699356 (494452 to 905186)	8185.98 (5783.06 to 10589.58)	−1.13 (−1.48 to −0.77)
Central Europe	1581070 (1006746 to 2194999)	8793.83 (5594.49 to 12213.26)	840262 (598732 to 1125067)	2743.77 (1954.88 to 3674.17)	−4.1 (−4.31 to −3.88)
Central Latin America	373041 (257328 to 497712)	4210.56 (2901.34 to 5621.6)	574863 (390415 to 796037)	1923.41 (1305.71 to 2662.82)	−2.73 (−2.87 to −2.59)
Central Sub-Saharan Africa	164151 (118258 to 224575)	7319.55 (5279.99 to 9941.52)	300225 (201584 to 414588)	5844.38 (3917.41 to 8073.88)	−0.93 (−1 to −0.86)
East Asia	3571201 (2711204 to 4461013)	4320.76 (3290.16 to 5378.52)	9627255 (6868386 to 12515275)	3878.61 (2768.44 to 5027.31)	0.12 (−0.29 to 0.54)
Eastern Europe	2956669 (1608379 to 4294099)	8948.87 (4862.57 to 13009.98)	1680509 (1015399 to 2513994)	3588.72 (2169.64 to 5371.67)	−3.64 (−4.19 to −3.09)
Eastern Sub-Saharan Africa	296538 (227213 to 375295)	3772.35 (2878.17 to 4774.79)	641594 (486307 to 793420)	3809.14 (2869.33 to 4726.84)	−0.14 (−0.23 to −0.06)
High-income Asia Pacific	228609 (60538 to 444562)	979.25 (256.23 to 1910.23)	277833 (154657 to 416497)	393.4 (220.72 to 584.32)	−3.1 (−3.42 to −2.78)
High-income North America	1209897 (454692 to 2157423)	2573.23 (966.95 to 4588.66)	341842 (156099 to 570322)	376.28 (171.95 to 627.17)	−6.8 (−7.2 to −6.4)
North Africa and Middle East	1736865 (1325163 to 2165726)	9835.74 (7482 to 12271.11)	3258683 (2464920 to 4085240)	6838.19 (5162.38 to 8568.96)	−1.19 (−1.26 to −1.12)
Oceania	22442 (16094 to 30596)	7594.33 (5458.06 to 10281.91)	49309 (34610 to 65700)	6664.34 (4679.98 to 8868.07)	−0.38 (−0.45 to −0.31)
South Asia	4146959 (3260875 to 5099711)	6751.52 (5296.39 to 8312.79)	11140635 (8588298 to 13618993)	6599.06 (5082.94 to 8069.97)	0.02 (−0.11 to 0.14)
Southeast Asia	1378621 (1055997 to 1724677)	5186.66 (3959.89 to 6495.53)	2529802 (1783483 to 3321163)	3495.04 (2467.2 to 4584.02)	−1.37 (−1.58 to −1.17)
Southern Latin America	193743 (105403 to 297993)	3485.36 (1897.27 to 5359.1)	106046 (61024 to 161329)	928.36 (534.59 to 1411.58)	−4.18 (−4.42 to −3.94)
Southern Sub-Saharan Africa	74539 (53970 to 96972)	2561.88 (1852.51 to 3334.99)	145423 (103132 to 190572)	2348.91 (1659.84 to 3082.32)	−0.3 (−0.76 to 0.16)
Tropical Latin America	348417 (197665 to 525573)	3512.92 (2003.34 to 5285.76)	308988 (173809 to 459113)	972.48 (547.09 to 1445.42)	−4.12 (−4.25 to −4)
Western Europe	2491755 (1232357 to 3946301)	3226.19 (1593.92 to 5115.39)	667610 (420415 to 934487)	481.84 (305.12 to 672.44)	−6.4 (−6.61 to −6.19)
Western Sub-Saharan Africa	523226 (395313 to 675872)	5645.26 (4260.61 to 7279.9)	1026590 (750692 to 1291597)	5361.84 (3934.4 to 6722.87)	−0.16 (−0.3 to −0.02)

IHD, ischemic Heart Disease; EAPC, estimated annual percentage change, SDl, Sociodemographic Index; Ul, uncertainty interval. EAPC is expressed as 95% CIs.

**Table 2:** The deaths cases and age-standardized deaths rate of IHD attributed to air pollution in 1990 and 2021, along with their temporal trend.

Region	Rate per 100 000(95%UI)				
	1990		2021		1990–2021
	Deaths cases	The age-standardized deaths rate	Deaths cases	The age-standardized deaths rate	EAPC
Global	1221853 (921412 to 1541964)	296.41 (221.15 to 376.96)	2005125 (1484963 to 2514494)	193.65 (143.12 to 243.06)	−1.41 (−1.51 to −1.31)
SDI region					
High SDI	271777 (166551 to 384367)	192.92 (117.76 to 273.59)	137862 (88732 to 188372)	43.57 (28.25 to 59.36)	−5.1 (−5.21 to −4.99)
High-middle SDI	378921 (268219 to 500825)	376.74 (264.38 to 499.8)	476499 (338048 to 621357)	196.4 (139.21 to 256.08)	−2.32 (−2.57 to −2.07)
Middle SDI	281073 (219059 to 344897)	311.92 (241.51 to 383.19)	715726 (527950 to 901298)	252.3 (185.77 to 317.8)	−0.53 (−0.71 to −0.34)
Low-middle SDI	215084 (169214 to 263262)	370.36 (290.28 to 454.12)	513785 (394662 to 631350)	345.25 (264.52 to 424.58)	−0.1 (−0.2 to 0)
Low SDI	72857 (57344 to 90695)	351.04 (274.94 to 436.62)	159504 (124233 to 195665)	345.92 (268.27 to 424.89)	0.1 (−0.02 to 0.23)
GBD region					
Andean Latin America	5794 (4341 to 7332)	270.9 (202.97 to 342.47)	6944 (4534 to 9886)	100.05 (65.34 to 142.37)	−3.67 (−3.95 to −3.39)
Australasia	1988 (69 to 5393)	68.88 (2.39 to 187.09)	1912 (1031 to 2921)	23.68 (12.77 to 36.17)	−4.16 (−4.66 to −3.66)
Caribbean	8877 (5541 to 13349)	304.97 (188.81 to 459.78)	11833 (7300 to 17168)	173.37 (107.16 to 251.16)	−1.78 (−2 to −1.56)
Central Asia	30783 (19353 to 44220)	627.52 (394.06 to 901.43)	40152 (28327 to 51968)	515.5 (363.27 to 666.81)	−1.04 (−1.35 to −0.73)
Central Europe	92475 (58967 to 128510)	559.48 (356.19 to 777.68)	55692 (39453 to 74700)	180.61 (127.93 to 242.31)	−3.93 (−4.15 to −3.72)
Central Latin America	21502 (14794 to 28652)	263.77 (181.24 to 351.9)	35635 (24070 to 49468)	122.12 (82.49 to 169.46)	−2.63 (−2.78 to −2.47)
Central Sub-Saharan Africa	7734 (5564 to 10555)	412.68 (297.42 to 558.77)	14838 (9916 to 20533)	338.08 (224.94 to 469.41)	−0.84 (−0.91 to −0.78)
East Asia	190421 (144970 to 237774)	285.04 (217.58 to 354.42)	623167 (445611 to 805402)	273.79 (195.62 to 352.81)	0.38 (−0.07 to 0.82)
Eastern Europe	176138 (95685 to 256680)	585.57 (317.49 to 854.24)	105740 (63565 to 158627)	229.45 (138 to 344.44)	−3.69 (−4.21 to −3.16)
Eastern Sub-Saharan Africa	14211 (10847 to 17988)	207.22 (156.6 to 263.04)	32551 (24554 to 40479)	218.38 (162.71 to 273.14)	0.02 (−0.07 to 0.1)
High-income Asia Pacific	14287 (3690 to 27960)	65.85 (16.75 to 129.4)	20458 (11244 to 31093)	25.2 (14 to 37.95)	−3.21 (−3.56 to −2.87)
High-income North America	76145 (28565 to 135926)	161.65 (60.64 to 288.59)	22313 (10154 to 37499)	23.9 (10.89 to 40.1)	−6.74 (−7.17 to −6.32)
North Africa and Middle East	90807 (68952 to 113297)	581.91 (439.67 to 726.39)	179872 (135281 to 225311)	416.77 (312.46 to 522.07)	−1.06 (−1.12 to −1)
Oceania	1040 (747 to 1415)	426.4 (306.4 to 574.65)	2386 (1673 to 3180)	373.65 (261.85 to 497.49)	−0.39 (−0.45 to −0.32)
South Asia	199158 (156370 to 244985)	366.54 (286.75 to 451.93)	579956 (446208 to 710218)	376.96 (289.46 to 462.01)	0.25 (0.08 to 0.42)
Southeast Asia	71714 (54855 to 89865)	303.48 (230.87 to 380.9)	136234 (96286 to 178745)	208.6 (147.62 to 273.55)	−1.31 (−1.52 to −1.1)
Southern Latin America	11552 (6306 to 17729)	222.12 (121.28 to 340.79)	6604 (3785 to 10107)	56.99 (32.68 to 87.18)	−4.23 (−4.49 to −3.98)
Southern Sub-Saharan Africa	4127 (2978 to 5399)	155.9 (112.15 to 204.11)	7792 (5478 to 10204)	141.65 (99.02 to 185.94)	−0.34 (−0.78 to 0.11)
Tropical Latin America	18627 (10610 to 27983)	209.15 (119.92 to 313.29)	16799 (9473 to 25090)	54.16 (30.54 to 80.93)	−4.27 (−4.37 to −4.16)
Western Europe	156825 (77291 to 249287)	204.51 (100.64 to 325.76)	48967 (30483 to 68696)	32.11 (20.12 to 44.92)	−6.21 (−6.44 to −5.97)
Western Sub-Saharan Africa	27645 (20885 to 35654)	335.48 (252.67 to 432.21)	55282 (40550 to 69256)	325.41 (239.41 to 406.34)	−0.08 (−0.21 to 0.05)

IHD, ischemic Heart Disease; EAPC, estimated annual percentage change, SDl, Sociodemographic Index; Ul, uncertainty interval. EAPC is expressed as 95% CIs.

**Table 3:** The YLDs cases and age-standardized YLDs rate of IHD attributed to air pollution in 1990 and 2021, along with their temporal trend.

Region	Rate per 100 000 (95%UI)				
	1990		2021		1990–2021
	YLDs cases	The age-standardized YLDs rate	YLDs cases	The age-standardized YLDs rate	EAPC
Global	400588 (227166 to 630209)	86.78 (49.33 to 136.16)	777285 (440728 to 1219129)	72.13 (40.91 to 113.06)	−0.6 (−0.68 to −0.53)
SDI region					
High SDI	81321 (42292 to 137892)	56.04 (29.14 to 95.12)	79453 (42406 to 128136)	27.67 (14.79 to 44.62)	−2.58 (−2.68 to −2.47)
High-middle SDI	117317 (65196 to 184272)	99.15 (55.22 to 155.34)	184071 (104392 to 288201)	72.22 (40.93 to 112.96)	−1.04 (−1.17 to −0.9)
Middle SDI	107587 (61646 to 169311)	98.77 (57.01 to 154.65)	278502 (159055 to 440722)	87.84 (50.26 to 138.84)	−0.28 (−0.41 to −0.15)
Low-middle SDI	69744 (40122 to 109606)	110.26 (63.78 to 172.46)	175731 (100978 to 276015)	109.01 (62.85 to 170.73)	0.02 (−0.05 to 0.09)
Low SDI	23941 (13792 to 37764)	103.63 (60.05 to 162.46)	58776 (33816 to 91924)	112.38 (65.03 to 174.82)	0.3 (0.26 to 0.33)
GBD region					
Andean Latin America	2536 (1431 to 3928)	112.11 (63.41 to 173.42)	5431 (2803 to 8828)	76.59 (39.55 to 124.47)	−1.48 (−1.65 to −1.3)
Australasia	574 (16 to 1596)	18.75 (0.51 to 52.27)	1371 (633 to 2406)	18.42 (8.51 to 32.34)	−1.04 (−1.5 to −0.57)
Caribbean	2793 (1318 to 5021)	89.08 (42.06 to 160.02)	4926 (2300 to 8359)	73.04 (34.12 to 123.86)	−0.67 (−0.8 to −0.53)
Central Asia	6296 (3097 to 10762)	119.78 (58.8 to 204.22)	9933 (5283 to 15871)	112.56 (59.93 to 179.64)	−0.39 (−0.51 to −0.27)
Central Europe	29280 (14643 to 49084)	157.09 (79.03 to 262.26)	26118 (14010 to 42828)	85.23 (45.74 to 139.88)	−2.03 (−2.28 to −1.78)
Central Latin America	8000 (4239 to 13120)	89.02 (47.34 to 145.67)	14990 (7953 to 24257)	50.01 (26.56 to 80.93)	−1.94 (−2.11 to −1.77)
Central Sub-Saharan Africa	1738 (1000 to 2743)	83.52 (48.35 to 131.69)	3766 (2134 to 5932)	76.09 (43.45 to 119.4)	−0.37 (−0.4 to −0.33)
East Asia	94050 (54039 to 147918)	100.18 (58.04 to 157.11)	260711 (151125 to 411064)	96.2 (55.83 to 151.65)	0.14 (−0.09 to 0.37)
Eastern Europe	38774 (17763 to 66864)	114.1 (52.35 to 195.89)	25001 (12497 to 42335)	53.15 (26.58 to 89.8)	−2.82 (−2.99 to −2.65)
Eastern Sub-Saharan Africa	7871 (4440 to 12529)	102.71 (58.31 to 162.8)	18768 (10567 to 29478)	109.94 (62.21 to 172.05)	0.24 (0.2 to 0.28)
High-income Asia Pacific	5392 (1170 to 11691)	22.21 (4.8 to 48.24)	12060 (6039 to 20885)	18.43 (9.3 to 31.77)	−1.02 (−1.38 to −0.65)
High-income North America	17052 (6090 to 33960)	35.92 (12.81 to 71.56)	9842 (3781 to 18483)	10.9 (4.19 to 20.47)	−4.73 (−5.1 to −4.36)
North Africa and Middle East	19432 (11141 to 30397)	109.35 (62.9 to 170.43)	54638 (31583 to 85150)	111.86 (64.79 to 173.87)	0.1 (0.01 to 0.19)
Oceania	290 (163 to 455)	104.3 (59.21 to 162.56)	723 (410 to 1137)	102.57 (58.46 to 160.99)	−0.02 (−0.08 to 0.04)
South Asia	67933 (39497 to 106057)	117.75 (68.83 to 182.86)	203371 (117090 to 318145)	121.83 (70.42 to 190.05)	0.18 (0.05 to 0.3)
Southeast Asia	26069 (14686 to 40844)	99.12 (56.23 to 154.78)	54170 (30144 to 87001)	75.25 (42.11 to 120.46)	−0.97 (−1.14 to −0.8)
Southern Latin America	2320 (1081 to 4062)	41.02 (19.12 to 71.88)	2929 (1399 to 5123)	25.71 (12.28 to 44.97)	−1.98 (−2.21 to −1.76)
Southern Sub-Saharan Africa	2618 (1458 to 4180)	88.15 (49.24 to 140.42)	4044 (2226 to 6517)	63.99 (35.43 to 102.79)	−1.17 (−1.33 to −1.01)
Tropical Latin America	7636 (3824 to 13436)	76.03 (38.27 to 133.32)	12189 (5692 to 21836)	38.52 (18 to 68.9)	−2.33 (−2.49 to −2.16)
Western Europe	51947 (22537 to 95060)	66.75 (28.93 to 122.37)	32057 (16078 to 53216)	24.69 (12.37 to 41.03)	−3.51 (−3.78 to −3.24)
Western Sub-Saharan Africa	7990 (4547 to 12565)	83.99 (48.01 to 131.82)	20247 (11532 to 32057)	101.72 (58.11 to 160.36)	0.74 (0.66 to 0.81)

IHD, ischemic Heart Disease; EAPC, estimated annual percentage change, SDl, Sociodemographic Index; Ul, uncertainty interval. EAPC is expressed as 95% CIs.

**Table 4:** The YLLs cases and age-standardized YLLs rate of IHD attributed to air pollution in 1990 and 2021, along with their temporal trend.

Region	Rate per 100 000(95%UI)				
	1990		2021		1990–2021
	YLLs cases	The age-standardized YLLs rate	YLLs cases	The age-standardized YLLs rate	EAPC
Global	21707400 (16569509 to 27201522)	4809.17 (3643.41 to 6057.29)	33786889 (25257005 to 42183966)	3170.09 (2366.06 to 3960.79)	−1.42 (−1.52 to −1.32)
SDI region					
High SDI	4309438 (2654079 to 6058496)	3002.65 (1844.72 to 4227.83)	1961301 (1286276 to 2665403)	662.27 (437.32 to 898.14)	−5.19 (−5.3 to −5.08)
High-middle SDI	6413034 (4572934 to 8453220)	5721.18 (4055.8 to 7559.66)	7221463 (5132103 to 9439931)	2900.49 (2059.94 to 3790.46)	−2.48 (−2.76 to −2.19)
Middle SDI	5215123 (4077742 to 6402485)	4963.28 (3864.01 to 6094.85)	11952841 (8851617 to 15054643)	3900.42 (2883.71 to 4912.71)	−0.68 (−0.85 to −0.51)
Low-middle SDI	4252643 (3352518 to 5200309)	6500.36 (5112.85 to 7957.37)	9586110 (7381152 to 11764870)	5907.44 (4541.12 to 7254.9)	−0.22 (−0.31 to −0.14)
Low SDI	1480545 (1166160 to 1845387)	6200.36 (4872.09 to 7720.7)	3036705 (2369682 to 3723829)	5836.92 (4543.13 to 7162.59)	−0.12 (−0.21 to −0.03)
GBD region					
Andean Latin America	96014 (71899 to 121884)	4271.04 (3199.71 to 5416.77)	107848 (70377 to 154096)	1529.24 (997.93 to 2184.26)	−3.79 (−4.07 to −3.51)
Australasia	32401 (1119 to 87907)	1075.1 (37.16 to 2919.48)	24746 (13361 to 37800)	322.32 (174.06 to 492.26)	−4.63 (−5.17 to −4.08)
Caribbean	154322 (98910 to 228852)	5002.49 (3186.74 to 7436.23)	203025 (127442 to 291333)	3007.19 (1889.74 to 4311.37)	−1.62 (−1.85 to −1.4)
Central Asia	515308 (323432 to 740693)	9871.39 (6195.67 to 14184.17)	689423 (487492 to 893828)	8073.42 (5702.59 to 10457.95)	−1.13 (−1.49 to −0.78)
Central Europe	1551790 (991933 to 2154822)	8636.74 (5515.04 to 11996.48)	814145 (580978 to 1090833)	2658.54 (1897.1 to 3562.39)	−4.15 (−4.36 to −3.93)
Central Latin America	365041 (251610 to 485876)	4121.54 (2838.11 to 5490.2)	559873 (380240 to 777500)	1873.39 (1271.93 to 2601.05)	−2.75 (−2.89 to −2.61)
Central Sub-Saharan Africa	162412 (116783 to 222642)	7236.03 (5210.78 to 9851.27)	296459 (198459 to 410326)	5768.29 (3851.37 to 7990.2)	−0.94 (−1.01 to −0.87)
East Asia	3477151 (2639152 to 4355909)	4220.58 (3213.03 to 5266.27)	9366544 (6686485 to 12187096)	3782.42 (2700.52 to 4905.76)	0.12 (−0.3 to 0.54)
Eastern Europe	2917895 (1585694 to 4245103)	8834.77 (4796.41 to 12862.53)	1655508 (997856 to 2480371)	3535.58 (2132.06 to 5300.22)	−3.65 (−4.21 to −3.09)
Eastern Sub-Saharan Africa	288668 (220692 to 366008)	3669.64 (2791.42 to 4654.87)	622827 (472500 to 772092)	3699.2 (2786.61 to 4601.91)	−0.15 (−0.24 to −0.07)
High-income Asia Pacific	223217 (59138 to 433489)	957.04 (250.51 to 1865.02)	265773 (147715 to 399382)	374.98 (210.21 to 558.49)	−3.17 (−3.49 to −2.85)
High-income North America	1192845 (447485 to 2126878)	2537.32 (951.81 to 4524.19)	332000 (151713 to 553814)	365.38 (167.11 to 608.83)	−6.84 (−7.24 to −6.44)
North Africa and Middle East	1717433 (1307960 to 2141965)	9726.39 (7385.14 to 12135.68)	3204045 (2415661 to 4016411)	6726.33 (5062.93 to 8428.78)	−1.21 (−1.28 to −1.14)
Oceania	22152 (15864 to 30269)	7490.03 (5377.28 to 10160.95)	48587 (34043 to 64825)	6561.77 (4600.26 to 8745.25)	−0.39 (−0.45 to −0.32)
South Asia	4079026 (3209591 to 5010728)	6633.77 (5207.66 to 8161.11)	10937264 (8426367 to 13389049)	6477.23 (4984.51 to 7932.55)	0.01 (−0.11 to 0.14)
Southeast Asia	1352552 (1038597 to 1693016)	5087.54 (3892.26 to 6375.29)	2475632 (1746428 to 3251353)	3419.8 (2415.41 to 4488)	−1.38 (−1.58 to −1.18)
Southern Latin America	191424 (104014 to 294436)	3444.34 (1873.02 to 5295.58)	103117 (59423 to 157065)	902.65 (520.52 to 1374.33)	−4.22 (−4.46 to −3.98)
Southern Sub-Saharan Africa	71921 (52035 to 94083)	2473.72 (1786.72 to 3236.78)	141379 (99823 to 184837)	2284.92 (1607.19 to 2992.27)	−0.28 (−0.74 to 0.19)
Tropical Latin America	340781 (192549 to 514097)	3436.88 (1952.27 to 5171.59)	296799 (167776 to 441692)	933.96 (527.94 to 1390.28)	−4.18 (−4.3 to −4.06)
Western Europe	2439808 (1203499 to 3863158)	3159.44 (1556.83 to 5008.21)	635552 (400292 to 886984)	457.14 (289.88 to 636.02)	−6.5 (−6.7 to −6.29)
Western Sub-Saharan Africa	515236 (389146 to 666408)	5561.27 (4195.03 to 7181.54)	1006343 (734067 to 1267326)	5260.12 (3851.04 to 6599.37)	−0.18 (−0.32 to −0.04)

IHD, ischemic Heart Disease; EAPC, estimated annual percentage change, SDl, Sociodemographic Index; Ul, uncertainty interval. EAPC is expressed as 95% CIs.

### 3.2 Burden of IHD attributed to AP across various subgroups (sex, age, SDI, GBD region, and country) and trends in disease burden

**Figure 2:**
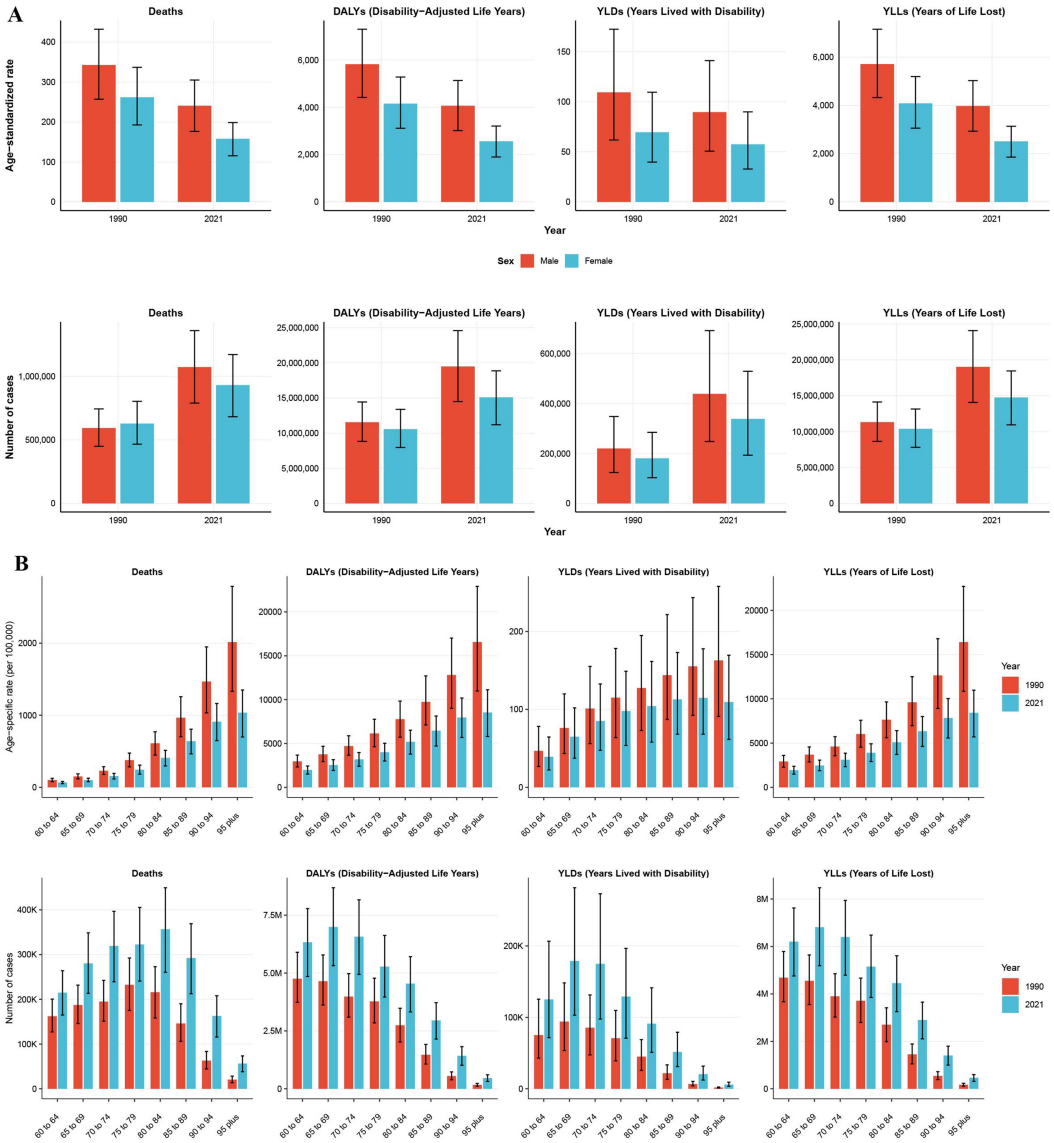
The number and age-standardized rates of various indicators reflecting the impact of air pollution on ischemic heart disease in people aged 60 years and older in 1990 and 2021 **(A)** and by age subgroups **(B)**. Error bars indicate 95% confidence intervals (CIs) for the estimated values based on GBD data.

**Figure 3:**
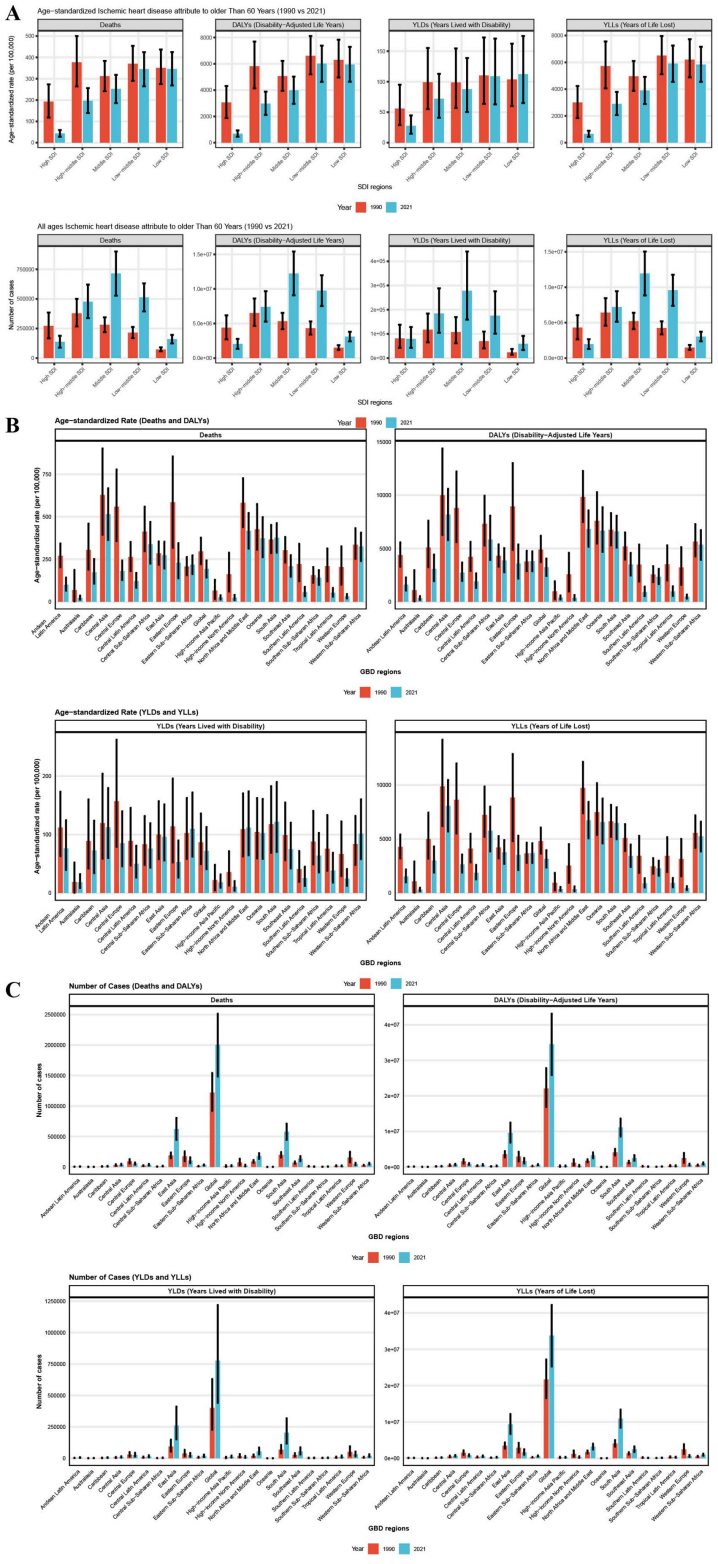
The number and age-standardized rates of various indicators reflecting the impact of air pollution on ischemic heart disease in people aged 60 years and older in 1990 and 2021 by SDI region subgroups **(A)** and GBD region subgroups **(B,C)**. Error bars indicate 95% confidence intervals (CIs) for the estimated values based on GBD data.

Between 1990 and 2021, both the burden of IHD attributed to AP and the ASRs were consistently higher in men than in women, with this gender disparity remaining largely unchanged over the period. In 2021, the disease burden ratios for men relative to women were 1.29:1 for DALYs, 1.15:1 for deaths, 1.3:1 for YLDs, and 1.29:1 for YLLs (Figure 2A). Regarding age groups, higher ASRs were observed in older age categories in 2021, although the 85 + age group exhibited a relatively lower disease burden compared to other age groups. From 1990 to 2021, the burden of disease increased across all age groups, while ASRs showed a general decline, with the most significant decreases occurring in older age groups (Figure 2B; Supplementary Figure S1A). With respect to the Socio-Demographic Index (SDI), disease burdens rose in all regions except those with a High SDI, where a decrease in burden was noted. This trend was especially prominent in the densely populated Middle and Low-Middle SDI regions, which together accounted for 63.6% of DALYs, 61.3% of deaths, 58.4% of YLDs, and 63.7% of YLLs. ASRs decreased across all SDI regions, with higher SDI regions experiencing more pronounced reductions, while Low SDI regions saw more limited declines. These changes were significantly correlated with the healthcare infrastructure and public awareness in each region (Figure 3A). As for health indicators, DALYs and YLLs remained at elevated levels over an extended period, suggesting that AP-induced IHD primarily results in health loss rather than a marked reduction in life expectancy (Supplementary Figures S1B,C).

In 2021, among Global Burden of Disease (GBD) regions, East Asia recorded the highest number of deaths and YLDs, while South Asia had the highest number of DALYs and YLLs, contributing to 31.1, 33.5, 32.2, and 32.4% of the global disease burden, respectively. Central Asia, North Africa, the Middle East, South Asia, and Oceania had the highest ASRs for disease burden, with disparities of more than 20-fold compared to the lowest regions, such as Australasia and High-Income North America. Between 1990 and 2021, except for a few Low SDI regions like East Asia, South Asia, and Eastern Sub-Saharan Africa, where the Age-Standardized Rates (EAPCs) for ASRs continued to rise, most regions saw a general decline, with High-Income North America and Western Europe experiencing the steepest decreases. The SDI regions showed the most rapid declines, with EAPCs for DALYs, deaths, YLDs, and YLLs in these regions at −6.8 (95% CI: −7.2 to −6.4), −6.4 (95% CI: −6.61 to −6.19), −6.74 (95% CI: −7.17 to −6.32), and −6.21 (95% CI: −6.44 to −5.97), respectively (Figure 3B; Tables 1–4).

**Figure 4:**
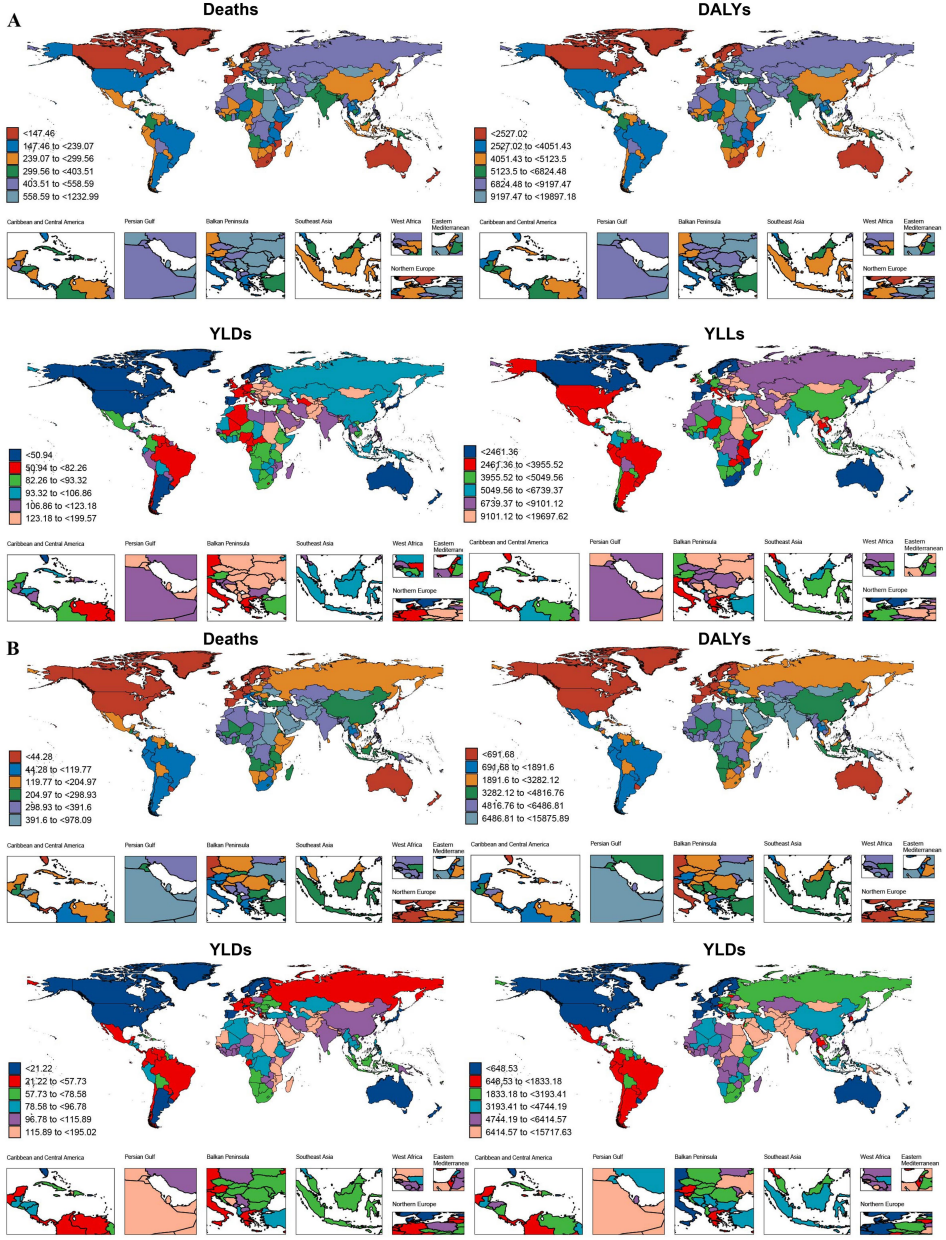
The age-standardized rates **(B)** of various indicators reflecting the impact of air pollution on ischemic heart disease in people aged 60 years and older in 1990 **(A)** and 2021 **(B)** from 204 countries and territories. DALYs-disability-adjusted life years, YLDs-years lived with disability, YLLs-years years of life lost.

In 2021, among 204 countries worldwide, China, India, Pakistan, Indonesia, and Egypt exhibited the highest disease burden, particularly in terms of DALYs and YLLs. Russia, with a significant number of deaths, ranked fourth globally, while Bangladesh reported the highest number of YLDs, following China and India. Although China and India had comparable DALYs, China’s deaths and YLDs were 1.3 and 1.6 times greater than those of India. Collectively, these two countries accounted for 53.3, 53.8, 52.9, and 53.4% of the global DALYs, deaths, YLDs, and YLLs, respectively. The ASRs for each disease burden indicator exhibited considerable variation between countries, with differences reaching up to 120-fold. For instance, Egypt’s Age-Standardized Death Rates (ASDRs) were as high as 15,876.89/100,000 (95% Uncertainty Interval [UI]: 11,637.94-20,532.18), while Iceland’s were just 137.08/100,000 (95% UI: 23.61–305.63). Similarly, Egypt’s YLLs had ASRs of up to 15,717.63/100,000 (95% UI: 11,477.1-20,344.03), while Iceland’s were only 132.00/100,000 (95% UI: 22.58–295.79). Countries such as Egypt, Afghanistan, Vanuatu, Solomon Islands, Uzbekistan, Sudan, Haiti, Yemen, and Tajikistan all had ASDRs greater than 1/10, over three times the global average, with corresponding ASRs for deaths and YLLs exceeding 600/100,000 and 0.9/10, respectively. For YLDs, global variations among countries were less pronounced, with the highest difference being approximately 40-fold. Kuwait, Qatar, Saudi Arabia, Sudan, and Bahrain had the highest ASRs, indicating that the management of AP-induced IHD in these countries requires particular attention. Additionally, we observed that the ASRs for each indicator in China were higher than the global average but notably lower than those in India (Figure 4; Tables 1–4).

### 3.3 Changes in ASR trends in IHD attributed to AP by SDI–based region and country

**Figure 5:**
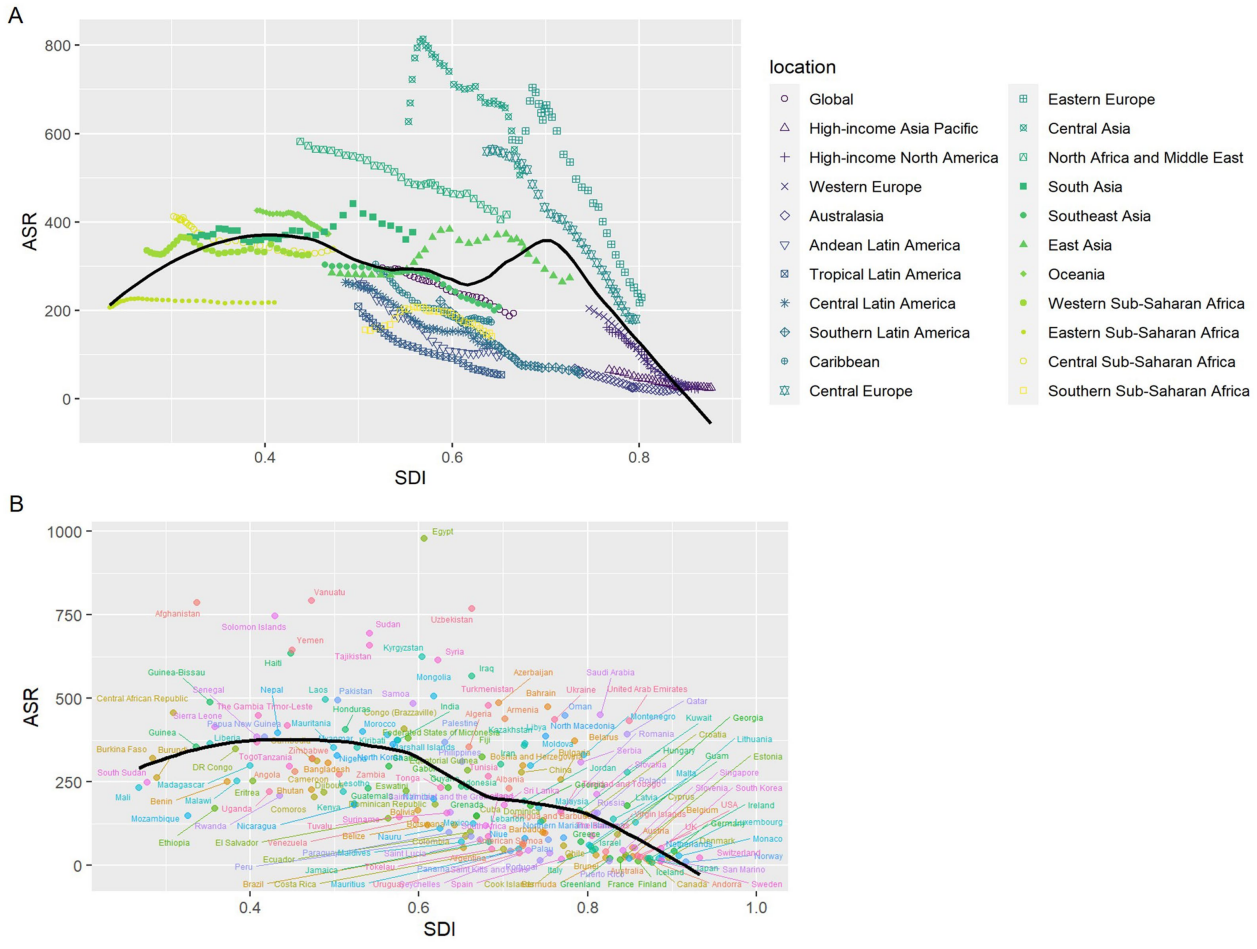
The age-standardized deaths rates trends in IHD attributed to AP by socio–demographic index, 1990–2021: **(A)** 21 GBD regions; **(B)** 204 countries and territories. Expected values for sociodemographic indices and disease deaths rates based on all sites are shown as black lines. ASR, age–standardized rate; SDI, socio–demographic index.

From 1990 to 2021, the trends in age-standardized death rates of IHD attributed to AP varied noticeably across different SDI regions. In Central Asia, we observed a clear reversal in the ASDR trend, suggesting a shift in disease burden during the study period. Regions like Southern Sub-Saharan Africa, East Asia, and South Asia showed large fluctuations over time, indicating unstable patterns. In low-SDI countries, there was no strong correlation between SDI and ASR. However, in high-SDI regions, the expected negative association was evident—higher SDI levels were generally linked to lower ASR. Middle-SDI regions also showed a similar inverse trend, though it was less consistent compared to high-SDI areas (Figure 5A).

At the national level in 2021, most countries followed the anticipated negative relationship between SDI and ASR, with only a few outliers. Among these, Egypt had an unusually high ASR that significantly exceeded what would be expected based on its SDI level (Figure 5B).

### 3.4 2036 projections

**Figure 6:**
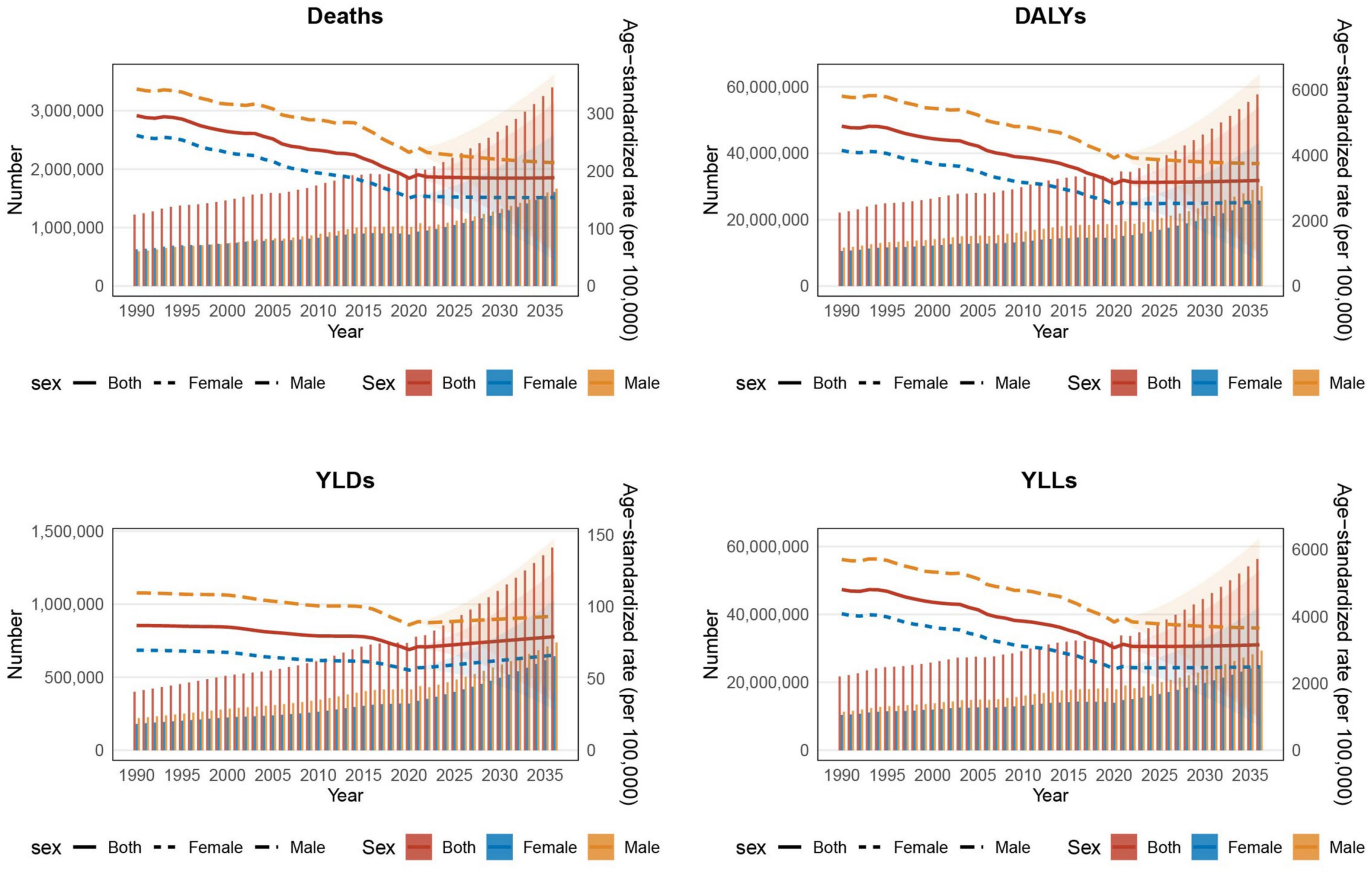
The trends of various indicators over different years, reflecting the impact of air pollution on ischemic heart disease in people aged 60 years and older by sex subgroups from 2022 to 2036 predicted by Bayesian age-period-cohort model. The left Y-axis represents the number of the total disease burden attributable to each indicator, The right Y-axis represents the age-standardized rate of the total disease burden attributable to each indicator, whereas the X-axis represents the years. DALYs-disability-adjusted life years, YLDs-years lived with disability, YLLs-years years of life lost. The shaded area represents the 95% confidence interval (CI) of the age-standardized rate.

To inform global strategies for the prevention and treatment of IHD caused by AP, we employed the BAPC model to forecast future trends in the global disease burden of IHD among individuals over 60 years of age. The projections suggest that in the coming decade, all disease burden indicators associated with AP-induced IHD will experience substantial increases, with growth rates surpassing 50%. Notably, YLDs may approach a one-fold increase. The corresponding ASRs are expected to shift from a steep decline to a more gradual reduction, with ASRs for YLDs potentially beginning to rise. These trends are anticipated to show minimal gender-based differences, although males will continue to experience higher rates than females (Figure 6).

## 4 Discussion

Previous studies mainly discussed the impact of PM2.5 on cardiovascular diseases (28), did not fully include other air pollution risk factors, or were based on the conclusions drawn from the old GBD database (29), and did not discuss the impact of AP on IHD disease burden in the older adult population in detail. To our knowledge, this study represents the first global analysis of the disease burden of IHD attributed to AP in individuals aged 60 and older. Our findings indicate that although the ASRs of AP related IHD have generally declined worldwide, the absolute numbers continue to rise, and this trend is likely to accelerate in the future, particularly for YLDs, which is concerning. Additionally, our analysis reveals significant disparities in disease burden and trends across different age groups, genders, regions, and countries. Older age groups and males have higher ASRs, with Low SDI regions such as Central Asia, North Africa and the Middle East, South Asia, and Oceania, as well as countries like Egypt, showing notably higher ASRs. In contrast, regions with higher SDI, such as Australasia and High-Income North America, have experienced rapid declines in ASRs. The regional differences are striking, with East Asia and South Asia contributing significantly to the global burden, while countries like China and India account for more than half of the global IHD burden. These disparities suggest the presence of significant global health inequities, which may be related to local environmental policies, health infrastructure, and socio-economic situation.

Notably, this is a different pattern than previously reported, our results highlight significant differences in disease burden due to regional differences, including SDI regions, GBD regions, and 204 countries, meanwhile, we subdivided people over 60 years old into groups for each additional 5 years and compared gender differences, the disease burden has been analysed more precisely.

Over the past decades, medical and public health advancements in many regions have led to stricter AP regulations, particularly in high SDI areas, reducing the impact of AP on cardiovascular disorders (30). This may account for the overall decline in ASRs globally, but the rise in the absolute number of IHD cases in individuals aged 60 and older can be attributed to global population growth, aging, and increased life expectancy (31).

Our results indicate that men have a higher burden of AP-related IHD than women and that this may be due to higher exposure to pollution sources such as traffic, industry, and agriculture in certain social, cultural, and behavioral contexts (32). Additionally, men are also more prone to higher cardiovascular risks that may be due to smoking rates, alcohol consumption, and lifestyle factors (33). This reminds men should focus on reducing exposure to pollution sources and promoting healthier lifestyles, such as smoking cessation programs and alcohol consumption reduction.

The disease burden is very high among those aged 60 and older and is rising in nearly all age groups in this group. This shows that AP is having a larger impact on IHD and is presumably due to greater physiological decline with age and higher rates of chronic illnesses such as hypertension, hyperglycemia, and hyperlipidemia in older individuals (34). These findings highlight the need for strengthened preventive measures in older adults, including air pollution exposure reduction, better management of chronic diseases, and regular cardiovascular health monitoring to mitigate the impact of AP on IHD.

The disease burden variations between SDI regions are also closely correlated with local AP levels, health infrastructure, public health policies, and residents’ health awareness. Strong health systems, effective public health policies, tighter environmental policies, and improved air quality are responsible for greater decreases in ASRs in higher SDI regions (35). Low SDI regions are distinguished by increased AP levels, limited health resources, poor social support, decreased health expenditure, and weak public health management, all of which are responsible for a higher IHD burden (36).

There are also disparities existing in IHD burden on a national level. In India and China, for instance, there is a severe burden and it is believed to be resulting from increasing population aging, heavy IHD burden in individuals aged 60 and older, and poor AP control, health management, and medical resources (37). Industrialization, increased industrial waste, higher numbers of motor vehicles, and energy consumption patterns in these regions are also increasing this burden (38). From a policy perspective, our findings emphasize the urgent need for targeted interventions. In low SDI regions, where ASRs remain high, strategies such as improving air quality regulations and expanding cardiovascular health programs are crucial. Conversely, in high SDI regions, where ASRs have declined, efforts should focus on sustaining these improvements through continuous monitoring and preventive healthcare initiatives. By addressing these region-specific challenges, global efforts to mitigate the IHD burden attributed to AP can be more effective and equitable.

The BAPC projections reveal that in the decade ahead, AP-related disease burden for IHD will rise significantly on a worldwide level. This highlights the requirement for prompt public health action and is crucial for guiding global public health planning. Nevertheless, the BAPC projections remain sensitive to potential policy interventions, such as improvements in healthcare accessibility stricter or AP control measures. To enhance the robustness of these findings, future research should incorporate sensitivity analyses to account for alternative scenarios, such as different degrees of air improvement or different demographic transition trajectories.

The rising disease burden of IHD due to AP will be tackled by future health policies with increased priority on pollution control and management of cardiovascular disease in older adults in areas with a greater burden. Increased health intervention, improved health infrastructure, and health education will be key to addressing these challenges.

## 5 Conclusion

The findings of this study reveal that health infrastructure and public health intervention play a pivotal role in preventing AP-induced ischemic heart disease (IHD). Public health intervention in areas with high disease burden is important to address AP-induced IHD as a rising global health issue. With rising proportions of older adults, AP-related health risks among this age group become more pronounced. Therefore, health management for older adults and more focused health intervention are urgently necessary. Public health policy should provide special attention to reducing exposure to AP among men and lifestyle factor-targeted intervention among men to alleviate disease burden of IHD.

Apart from this, health infrastructure in low-SDI and developing nations requires strengthening with special focus on health management and AP control. Intervention will minimize disease burden in these regions. Projections for 2036 are that disease burden indicators will see a significant increase. Global public health policies should not only focus on mitigating AP but also improving health infrastructure and public health education to tackle future challenges.

In conclusion, this study employs the newest global data (GBD 2021) for populations across different SDI regions, GBD regions, and countries to make the results more representative on a global scale. The study focuses on the impact of AP on IHD burden in individuals aged 60 and older and provides an age-group-specific analysis. By concentrating on the older adult, this research offers a more precise understanding of the effects of AP on high-risk groups. Additionally, using the BAPC model to forecast future trends, this study provides valuable foresight for global IHD prevention and control strategies, with significant public health implications.

Although this study reveals the association between AP and IHD burden, it is important to note that the observational nature of the study design does not allow for direct causal inferences. While factors such as SDI and GBD regions were considered, differences in health policies, healthcare levels, and pollution control measures across countries may not be fully explained by these factors, warranting further in-depth analysis.

## Data Availability

The original contributions presented in the study are included in the article/Supplementary material, further inquiries can be directed to the corresponding authors.

## Footnotes

^1^ http://ghdx.healthdata.org/
