# Supplementary figures and images for: Global burden trends and future predictions of ischemic heart disease attributable to air pollution in people aged 60 years and older, 1990–2021
Source: Front Public Health. 2025 Jul 4;13:1598092. doi: 10.3389/fpubh.2025.1598092 (PMC12270858; doi:10.3389/fpubh.2025.1598092)

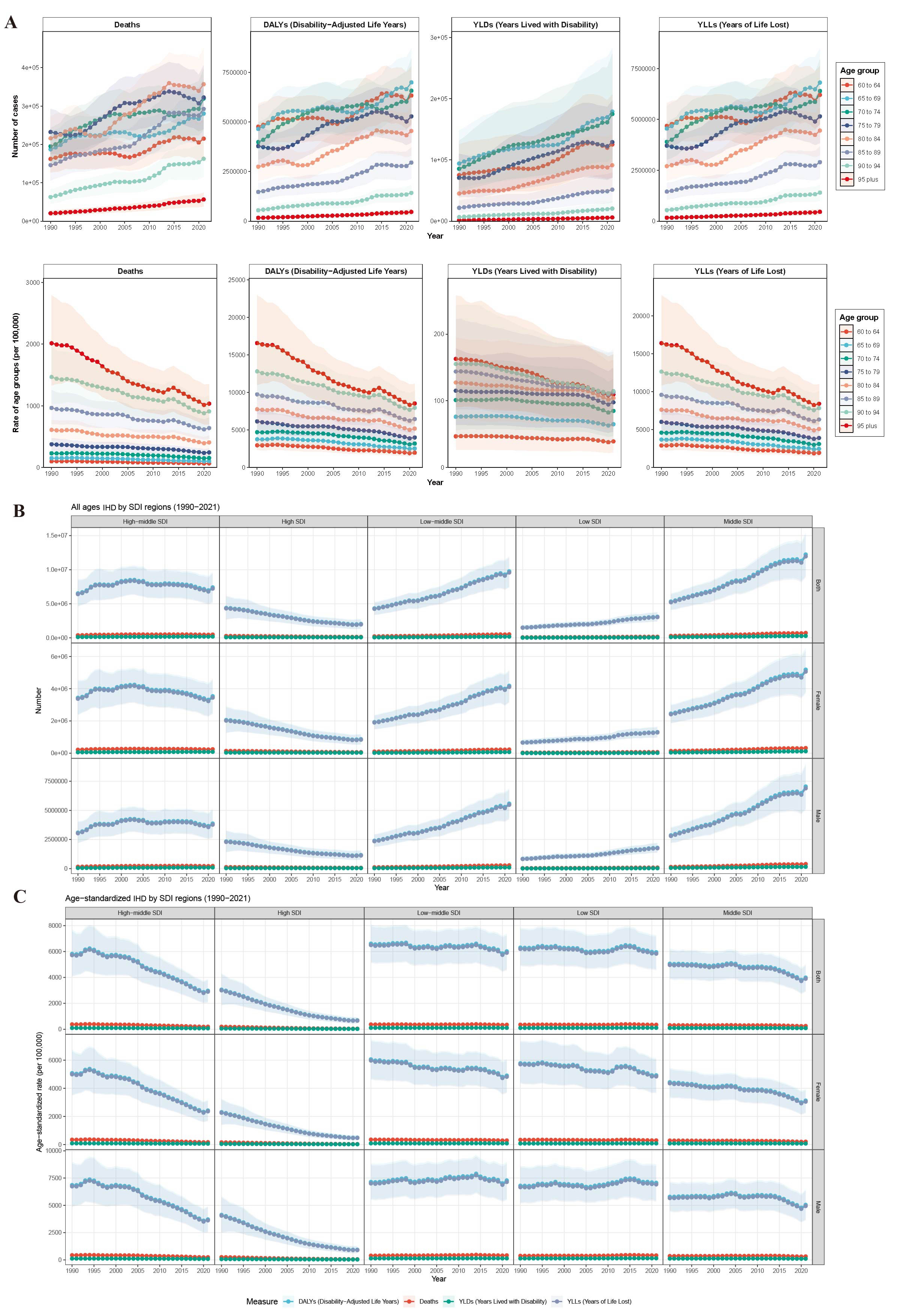

Supplement: Supplementary file 1 [file Image_1.JPEG]
